# How about Lunch? Consequences of the Meal Context on Cognition and Emotion

**DOI:** 10.1371/journal.pone.0070314

**Published:** 2013-07-31

**Authors:** Werner Sommer, Birgit Stürmer, Olga Shmuilovich, Manuel Martin-Loeches, Annekathrin Schacht

**Affiliations:** 1 Department of Psychology, Humboldt-Universität zu Berlin, Berlin, Germany; 2 General Psychology and Neurocognitive Psychology, International Psychoanalytic University, Berlin, Germany; 3 Center for Human Evolution and Behavior, UCM-ISCIII, Madrid, Spain; 4 Department of Psychobiology, Complutense University of Madrid, Madrid, Spain; 5 Courant Research Centre Text Structures, University of Göttingen, Göttingen, Germany; The University of Queensland, Australia

## Abstract

Although research addresses the effects of a meal’s context on food preference, the psychological consequences of meal situations are largely unexplored. We compared the cognitive and emotional effects of a restaurant meal eaten in the company of others to a solitary meal consumed in a plain office using pre- and post-tests analysis and controlling for the kind and amount of food consumed. Three tasks were conducted, measuring: (1) semantic memory (2) cognitive control and error monitoring, and (3) processing of emotional facial expressions. Covert processes in these tasks were assessed with event-related brain potentials. A mood rating questionnaire indicated a relaxation effect of the restaurant as compared to the plain meal situation. The restaurant meal increased sensitivity to threatening facial expressions and diminished cognitive control and error monitoring. No effects were observed for semantic memory. These findings provide the first experimental evidence that a restaurant meal with a social component may be more relaxing than a meal eaten alone in a plain setting and may reduce cognitive control.

## Introduction

Meals are of enormous importance to human beings not only for providing nutrition and energy but also as a cultural and social institution [1–3]. Moreover, meals are a major source of pleasure for almost all humans [4]. Scientific research on meals is largely driven by concerns about the impact of food on physical and mental well-being. In this regard – and from a psychological point of view – there is mounting evidence that one’s diet may influence cognition as well as emotional states. However, analysis of personal experience shows that meals seem to have more far-reaching effects. A nice meal, especially when taken in agreeable company, can put you in a good mood; it may foster new ideas, mitigate social tensions, and promote mutual agreement in business, politics, and families. In other words, a pleasant meal seems to elicit or modify emotional, cognitive, and social processes. Interestingly, these contextual effects of food consumption on psychological processes appear to be largely unexplored on a scientific level. It was the aim of the present study to narrow this gap by investigating cognitive and emotional consequences of a pleasant restaurant meal in company as compared to a plain, solitary meal, while controlling for quantity and quality of food consumption.

A sizeable body of research has shown contextual effects of the subjective experience of food, as reviewed by Meiselman [5]. A home meal leads to higher ratings of a food product than a standard laboratory environment [6]. The environment in which food is consumed is associated with the expected food quality, ranging from home and restaurant at the top over school and military canteens all the way down to airlines and hospitals [7]. Food consumed in an institutional setting is rated lower than in a restaurant setting [8, 9].

Although meals are considered to be a social activity in which humans engage [4], sociability (or commensality) during meals differs widely, with a much higher frequency of solitary meals in work than in private (often family) meal situations [10]. A social situation may also affect acceptability of certain meals [11]. Overall, these studies have shown that contextual effects are powerful determinants of the meal experience.

That meals have meaning and consequences beyond the proximal food-related experience is supported not only by every-day knowledge but also by scientific evidence. Danziger, Levav, and Avnaim-Pesso [12] reported that judges’ sentences become more severe as time since the last meal break lengthens. Unfortunately, it is not clear whether this is related to the meal (e.g. glucose availability) or to fatigue due to time on task. That meals have a special social significance has been indicated by a recent report by Kniffin and Wansink [13]: Sharing a meal with an opposite-sex friend can trigger more jealousy from one’s partner than going out for coffee with the same opposite-sex friend.

The present study aimed to investigate the effects of a meal situation on cognitive and emotional processes. While holding constant the kind and amount of food consumed, we contrasted two different every-day meal situations: eating food selected by others, alone, in a bland office environment, within a short time period versus dining in company in a restaurant with a choice of food and ample time for consuming the self-selected meal. In addition, the latter condition included a 15- to 20-minute walk from the restaurant to the lab where the experiments described below were performed; the office, in which the plain meal was consumed, was next door to the lab. Hence, the meal contexts differed in a number of variables that have been shown to be important at least as food preference and acceptability are concerned

Since meals can have effects on mood, emotion research may be a good starting point if one looks for possible distal, non-nutritional effects of meal contexts. It is widely accepted that emotions (or affects) modulate cognitive processes [14,15]. This kind of research commonly distinguishes between short-term emotions and longer-lasting moods [16]. In the present context, the effects of such longer-lasting mood states are most important. On the one hand, positive mood states have been considered to be advantageous for creativity [17] because they may – among others – enhance cognitive flexibility [16]. In a recent comprehensive review, Baas, De Dreu, and Nijstad [18] concluded that creativity is enhanced by positive mood states, especially when the mood is activating and “promotion”-focused rather than calm and relaxed. That positive mood enhances cognitive fluency (flexibility) has also been confirmed by some studies of executive functions, as reviewed by Mitchel and Philip [16]. Positive mood facilitates inferences based on general knowledge while a sad mood facilitates responses to primed stimuli [19]. Lyubomirsky, King, and Diener [20] proposed that “People in a positive mood are more likely to have richer associations within existing knowledge structures, and thus are likely to be more flexible and original. Those in a good mood will excel when the task is complex and past learning can be used in a heuristic way to more efficiently solve the task or when creativity and flexibility are required.” (p. 840). On the other hand, positive mood has been found to impair mechanisms of adaptive cognitive control like updating, planning, and switching in a number of other studies [16].

Investigating the neural concomitants of mood and emotional states can provide more direct access to the neurocognitive mechanisms underlying such effects. For instance, Federmeier, Kirson, Moreno, and Kutas [21] reported that the N400 component in the event-related brain potential (ERP) to semantically incongruous words in a sentence was reduced during a positive mood state. The authors concluded that mood states are associated with dynamic changes in how semantic memory is used on-line. This is in agreement with the facilitating effects of positive mood on cognitive flexibility and memory processes mentioned above.

Several studies indicate that mood states alter error processing. Findings from one of our labs show that the error-related negativity (Ne) – an ERP component observed shortly after an incorrect response – is largest in the context of positive feedback [22]. In line with these results, West and Travers [23] showed that participants in a happy mood and with low self-reported boredom showed the largest Ne. An increased Ne is often related to improved performance monitoring as a process of cognitive control [24].

The present study addressed the *general question* whether typical meal situations can affect cognitive and emotional processes – independent of the caloric and nutritional value of the food. These questions were investigated using a pre-post, between-group design. Two groups of female participants were tested on several experimental tasks, including EEG recordings in pre- and post-meal sessions. For the meal, the participants of the experimental group visited a restaurant accompanied by a friend where they had lunch of their choice to be consumed at leisure. A control group matched to the experimental group for gender, general food preferences, and body weight, ate the same kind and amount of food – controlling for nutrients and energy – but without the option of choice, alone in a plain office room without service and with only a minimum of time for the meal. The manipulation of meal selection, a restaurant setting and company allowed for several contextual factors that may potentially contribute to a pleasant meal context for the experimental group. In the control group, we attempted to reduce the meal and its context as much as possible to the intake of calories and nutrients but without inducing adverse emotional states. In line with previous findings about affective influences on cognition, we used three tasks to assess the influence of the meal context manipulation on psychological states and processes.

### Semantic memory

As discussed above, Federmeier et al. [21] reported diminished N400 amplitudes during pleasant mood for between-category semantic violations, presumably indicating facilitated search processes in semantic memory. Therefore, we employed a task that induces an N400 component: Semantically related and unrelated pairs of words were presented one after the other, requiring decisions about their relatedness. In this situation, the N400 component elicited by the second word is larger for semantically unrelated than related word pairs. This effect is explained by higher demands on semantic processes for the unrelated words (for a review see 25). In line with these findings and assuming that the restaurant meal would induce a positive mood, we predicted the N400 to be smaller for the experimental group (EG) in the post-meal session; in addition, response times (RTs) in the semantic decision task should be shorter because of increased fluency due to positive mood.

### Cognitive control and error processing

A Simon-type interference task with equally probable compatible and incompatible events was used to evoke cognitive conflicts and incorrect responses. Here we expected a lack of cognitive control in the EG, reflected in larger (in)compatibility effects in performance and the N2 component of the ERP, and diminished error processing as reflected in the error-related negativity (Ne [26]).

Usually, performance in trials where the response location does not correspond with the stimulus location is worse than in corresponding trials (the Simon effect). This is interpreted as a consequence of a conflict between different responses activated by stimulus location and stimulus shape, which takes time to be recognized and resolved before the correct response can be made. Recently, van Steenbergen and colleagues claimed that adaptive conflict control is reduced by reward and in positive mood states [27], [28]. We, therefore, hypothesized that the effect of the restaurant meal in the experimental group counteracts conflict control. The Simon effect – as a measure of cognitive conflict - should, therefore, be enlarged.

The Simon task also allowed assessing the processing of incorrect responses. The Ne, elicited by incorrect responses has been found to be amenable by mood and reward. If positive mood attenuates cognitive control [18], it might also adversely affect performance-monitoring processes. We therefore assumed that the Ne would be diminished in the EG relative to the control group (CG) in the post-meal session.

Following the Ne to incorrect responses there is a positive-going ERP component, the error positivity (Pe). The Pe has been related to the conscious perception of committing an error [29], [30]. We assumed that the restaurant meal would diminish conscious awareness of actions slips and thus reduce the Pe in the EG relative to the CG.

### Emotion Processing

In addition, we assessed the effects of the meal situation on the processing of emotional stimuli consisting of faces showing happy, angry, or neutral expressions. Previous studies have shown that emotional facial expressions involuntarily capture attention and recruit sensory resources as reflected in modulations of distinguishable ERPs components. The so-called early posterior negativity (EPN; [31]) typically starts around 150 to 200 ms after stimulus onset in response to emotional facial expressions [32] relative to neutral expressions. The EPN is interpreted as reflecting enhanced allocation of sensory resources at a perceptual stage of stimulus processing (for review 33).

The EPN is often followed by the increase of a centro-parietal positivity, termed late positive complex (LPC), in response to emotional expressions [32], [34]. The LPC has been suggested to reflect sustained, elaborative, and, possibly, conscious processing of emotional stimuli during later stages. Some reports have also shown a modulation of the face-sensitive N170 component by emotional expressions – particularly, by expressions of negative emotions. Other studies reported the N170 to be neither face-nor emotion-sensitive (for a recent overview, please see 35). We predicted that mood states induced by the restaurant meal situation might modulate effects of emotional facial expressions on ERP components. In line with the idea that positive mood increases cognitive flexibility, we specifically expected stronger effects of the emotional expressions on the EPN in the EG.

## Methods

### Participants

Thirty-two women were assigned in equal numbers to the experimental or control group (*M* = 24.75 vs. 23.75 years). Women were selected as participants to simplify the procedure and the interpretation of results. Most participants were students of the Humboldt-Universität zu Berlin; since they might have guessed the purpose of the study, Psychology students were not recruited. Twenty-nine participants were right-handed, the other three left-handed [36]. Participants of both groups were matched pair-wise for body mass index (BMI; *M* = 22.4 vs. 21.1) and weight (*M* = 62 vs. 60 kg). None of the participants had any special dietary restrictions, for example, vegan or macrobiotic diet; however, participants with ovo-lacto-vegetarian diets were accepted. The menu offered only vegetarian meals because many young women are vegetarians and it simplified the experimental matching. Furthermore, all participants reported no food allergies, no history of eating disorders, no history of depression (score in the German version of the Beck Depression Inventory, BDI [37] < 13), nor any other psychiatric or neurological disorders. Prior to the study, candidate participants were presented with a list containing all main ingredients of the meals on the offered menu. If any ingredient was disliked, the candidate was not included in the sample. Prior knowledge of the menu was not considered critical since this is common when the same restaurant is visited on a regular basis or if the menu is previewed on the Internet.

The food and drinks served during the study were provided for free and 8 euro/hour were paid for participation in the pre- and post-meal sessions. Written informed consent was obtained from all participants prior to the pre-meal session. The study was conducted in accordance with the Declaration of Helsinki and was approved by the local ethics committee of the Department of Psychology of the Humboldt-Universität zu Berlin. Participants were informed in writing that the aim of the study was to assess the effects of a meal on several psychological functions (word and face recognition, and reaction time). No mention was made of mood or emotions.

### Procedure

All participants took part in each of three study phases: (1) pre-meal session, (2) meal session, and (3) post-meal session. Pre- and post-meal sessions involved the same tasks (on a different set of items) and were always initiated at 1 pm. The pre-meal and meal sessions were separated by a minimum of one week but no more than two weeks; the meal session immediately preceded the post-test session. Participants were advised to have enough sleep in the nights preceding the test sessions, not to consume unusual amounts of alcohol in the evenings before the test and to have breakfast in the morning of the testing days in accordance with their usual habits.

#### Pre-meal session

The pre-meal session started by measuring body weight and height. Next, the handedness questionnaire [36] and the BDI-II [37] were completed. After the electrodes for EEG recording had been applied, the Multidimensional Mood State Questionnaire (MDBF [38]) short version “A” was administered. At the end of the session, the MDBF short version “B” was completed.

#### Meal session

On the day of the meal and post-meal session, participants were to refrain from eating and consumption of alcoholic beverages for four hours prior to the start of the experiment.

Each participant in the Experimental Group (EG) was asked to invite a companion of her liking for lunch. At noon she went with her companion to a medium-size Italian restaurant run by an Italian family who agreed with the arrangement of a restricted menu for our participants. In this restaurant, soft background music plays and guests are served by a waiter. The participants in the study were being served during lunchtime while other customers were present in the restaurant as well. Apart from the restriction of the menu and the “doggy bag” treatment of the left-overs (see below), the participants in our study were treated like the other customers. The restricted menu for the present study consisted of a selection of vegetarian meals taken from the standard menu and offered 7 pizzas, 12 pasta dishes, and 3 different non-alcoholic beverages, not including cola or coffee because of the coffein contents. Each participant and her companion were free to choose any of the aforementioned dishes and drinks. They were allowed 60 minutes for dining and were encouraged to eat at leisure. If the participant did not finish any part of the meal, it was placed in a doggy bag to be taken to the lab where it was weighed by the experimenter. Immediately after completing the meal, the participant walked to the lab (15 to 20 min), where the post-meal session of the study was conducted without further delay. All participants completed at least half of their meal.

Each participant of the Control Group (CG) received the same meal as her matched EG partner; that is, for the CG there was no choice of food. The meal was to be picked up by the CG participant from the take-away counter of the same restaurant were the EG had lunch. The participant had to bring the meal to the lab, where she was met by the experimenter. She was then shown to a small office room, in which she consumed the take-away food. If the matched experimental participant had not finished her meal, the size of the meal of the control participant was reduced – monitored with the help of an electronic scale – in order to equate the amount of food consumed by the matched participant pairs. The CG participants had 20 min to consume their meals, which is a very common duration of meals [5]. Unless the participant indicated finishing their meal would be uncomfortable, participants were asked to eat all of the food served to them. If a CG participant was unable to finish at least 60% of the meal, she was excluded from the study and replaced by a different matched person, to ensure that participants from each CG EG pair ate approximately the same amount of food. The room in which the meal was eaten was a standard office (ca. 12 m^2^) with plain office furniture and without decoration. During the meal no other person was present. No music or other media were allowed.

#### Post-meal session

The post-meal session took place immediately after the meal (CG) or arrival at the lab (EG). Except for the handedness and depression questionnaires, the post-meal session proceeded in the same way as the pre-meal session (except that now the order of the mood questionnaire versions was reversed). The order of tasks within the test sessions was fixed for all participants. For all tasks, stimulus response assignments were counterbalanced across participants.

### Questionnaires

Mood states were assessed with the Multidimensional Mood State Questionnaire (MDBF) short version “A” and “B” [38]. The MDBF short version consists of 12 questions to be answered on 5-point Likert scales (from “definitely not” to “very much”) and yields scores on the scales GS “bad to good mood”, WM “sleepy to awake”, and RU “restless to calm” (range of scores on all scales: 4 to 20).

Depression states were assessed with the German version of the BDI-II [37]; the scale of the BDI-II ranges from 0 to 63; scores <13 are considered as normal. A short questionnaire with Likert-scale items about the restaurant’s service quality (4 items) and atmosphere (6 items) for the experimental group and about meal tastiness (1 item) for both groups was also administered.

### Semantic Memory Task

Word stimuli were taken from Dimigen, Kliegl, and Sommer [39] and consisted of 100 pairs of semantically related nouns. Unrelated pairs were constructed by randomly reassigning the words into new pairs. For related and unrelated pairs two sets each of 50 pairs were made for the pre- and post-meal sessions, respectively.

Participants were presented with pairs of nouns; the first noun (prime) was either semantically related or unrelated to the second noun (target). Each trial started with a fixation cross presented for 500 ms, followed by the prime for 250 ms. After 200 ms of blank screen, the target was presented until a response occurred or for 1 s if no response was given until then. The next trial started after the presentation of a blank screen for a 1 second duration. Participants indicated whether the presented word was similar in meaning to the previous word (prime) by pressing a button with either their right or left hand. There was no feedback about correctness of the judgment. This task lasted for approximately 7 minutes, including four practice trials.

### Cognitive control (Simon) task

Stimuli consisted of two shapes (square and diamond). The maximal diameter was 0.75° visual angle each. Two response buttons were placed behind each other at a distance of 10 cm on the table in front of the participants in their mid-sagittal line.

Each trial started with a fixation point followed by a square or a diamond, presented 1 degree above or below fixation for 200 ms. Participants were to respond to the square and diamond shapes by pressing the distal or proximal response button with the left or right index finger, respectively. No feedback was given about response correctness. The stimulus-response key and the finger-key mappings were counterbalanced. The next trial started after a constant response-to-stimulus interval of 1.5 s. The task took about 20 min and started with 36 training trials followed by 9 experimental blocks of 69 trials each, separated by short rests. Compatible (311 trials) and incompatible conditions (310 trials) were randomized.

### Emotion processing task

For each of the face tests in the pre- and post-meal sessions, three sets of 50 unfamiliar faces of young adults with happy, neutral, or angry emotional facial expressions were assembled (taken from [40, 41]). Half of the faces in each set showed females, the other half males.

Faces were presented in random order. After 500 ms of fixation a face was presented for 1s, followed by a blank screen for 500 ms. Participants had to make timed decisions on the gender of the face by button presses with left and right index fingers and did not receive feedback about response correctness.

### Electrophysiological recordings

The EEG was recorded from 32 Ag/AgCl electrodes (impedances < 5 kOhm) with a band-pass of 0.016 to 70 Hz at a sampling rate of 250 Hz using a Brain Amp DC amplifier (Brain Products GmbH, Munich, Germany). All signals were initially referenced to an electrode placed on the left mastoid (A1). Offline, the EEG data was converted to average reference and low-pass filtered at 30 Hz. Trials with incorrect or missing responses were excluded from further analysis. Epochs containing artifacts were automatically discarded if in any channel showed a voltages in excess of -100 or +100 μV or voltage step > 50 μV between two adjacent sampling points.

All statistical analyses were conducted using PASW Statistics 18.The analyses of error rates, reaction times, and ERP amplitudes were performed with analysis of variance (ANOVA) including a within factor group and repeated measures on session (pre- vs. post-meal), compatibility (compatible, incompatible) – for the Simon task, and – for ERP amplitudes – electrode. If all recording sites are entered as levels of factor electrode, the effects of all other factors can only be interpreted if they interact with electrode because the average reference centers all amplitudes within a given cell at zero. For the analysis of meal-induced mood effects, a factor beginning versus end of session was added. Except for specific a-priori hypotheses, all post-hoc comparisons were corrected using the Bonferroni method. In case of sphericity violations, all ANOVAS were Huynh-Feldt corrected.

## Results and Discussion

### Questionnaires

#### Questionnaire about restaurant and food

On a scale from 1 (very good) to 6 (deficient), the EG rated the restaurant as “good” (*M* = 1.86) and the meal quality as “good to very good” (*M* = 1.62); the CG rated the meal quality as “good” (*M* = 2.19), which did not differ significantly from the EG (*t* = -1.4). Numerically – but not statistically - the same meals were rated somewhat lower by CG participants, possibly owing to the cooling of the food during the 15-min transport or to the lack of free choice.

#### Mood

Given the possible range of scores in the MDBF from 4 to 20, the GS scale showed that mood tended to be quite positive overall but showed a slight decline from the beginning to the end of sessions as a main effect (*M* = 17.8 vs. 16.6), *F*(1,15) = 22.4, *p* < .001. During the sessions there was decreasing wakefulness on the WM scale (*M* = 13.8 vs. 11.1), *F*(1,15) = 127.1, *p* < .001, and an increasing restlessness on the RU scale (*M* = 17.5 vs. 15.8), *F*(1,15) = 37.7, *p* < .001. The decreasing wakefulness over the sessions was more pronounced in the pre-meal than in the post-meal session (Mean Diff. = 3.44 vs. 2.16), *F*(1,15) = 5.4, *p* < .05. It is important to note the absence of a significant interaction of meal context (factor Group) with Session in any of the three scales. None of the three individual scales showed an effect due to the restaurant meal as compared to the plain meal.

Interestingly, when considering only the mood ratings at the beginning of each session, the EG demonstrated both an increase of calmness on the RU scale (from *M* = 17.0 to 18.1) and a decrease of wakefulness on the WM scale (from *M* = 14.37 to 13.13). This combination of increased calmness and decreased wakefulness might be conceived as relaxation effect. In an attempt to test this relaxation effect, we performed a *t*-test on the difference between the scores on the RU and WM scales. There was a significant increase of this “relaxation score” from pre-meal session to the post-meal session, *t*(1,15) = -2.14, *p* = .049) for the EG; whereas for the CG, the difference failed significance by a wide margin, *t*(1,15) = -0.150, *p* = .883).

### Semantic Memory

#### Performance

From pre-meal to post-meal session error rates increased (*M* = 4.9 vs. 7.6%), *F*(1,15) = 6.9, *p* < .05, whereas RTs decreased (*M* = 780 vs. 685 ms), *F*(1,15) = 28.5, *p* <. 001. Main effects of priming were significant in error rates (*M*(primed–unprimed) = -7.8%), *F*(1,15) = 27.6, *p* < .001, but not in RTs (*M*(primed–unprimed) = -5.7 ms), *F*(1,15) < 1.

#### ERPs

**Figure 1:**
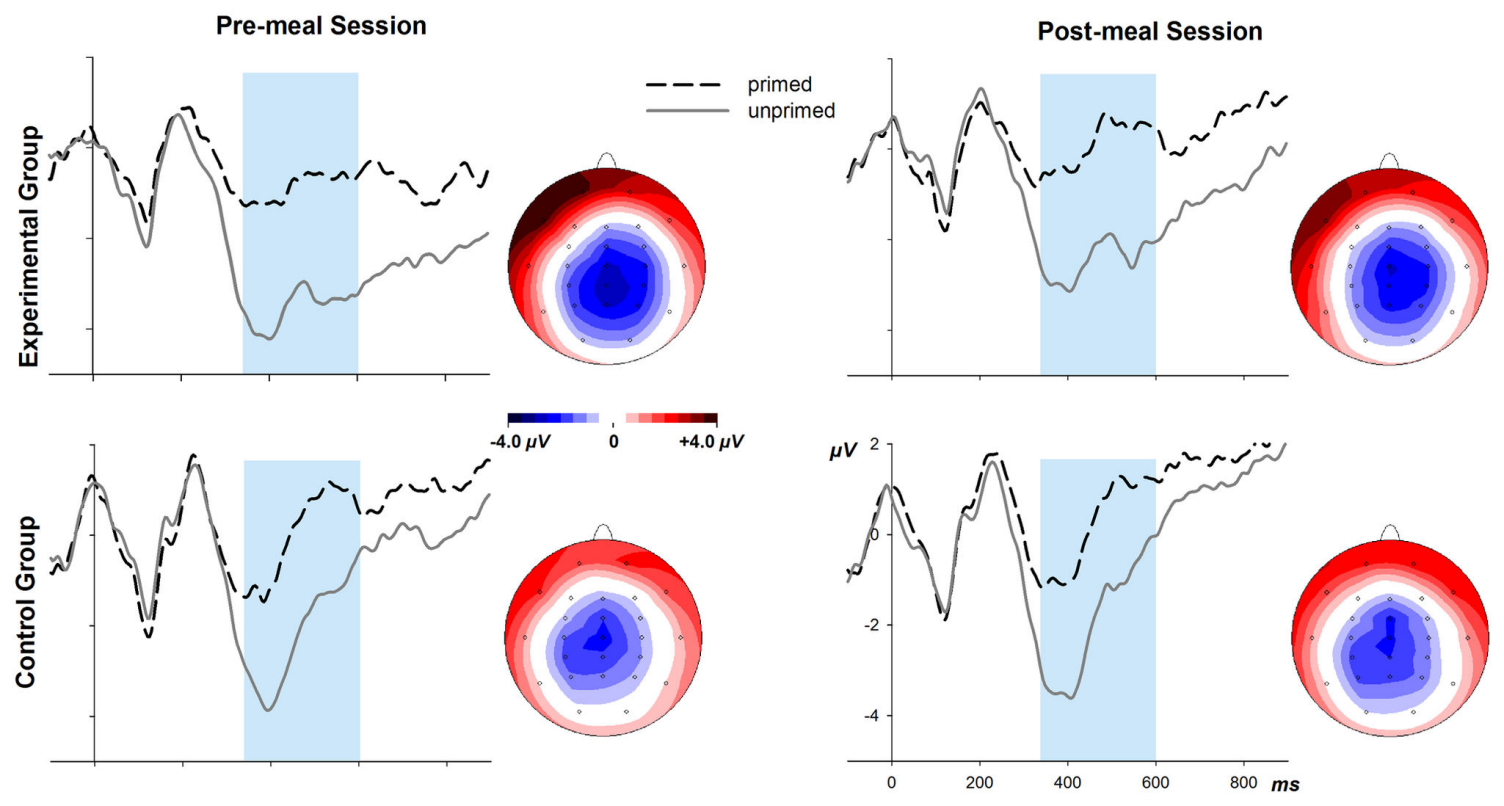
ERPs from the semantic memory task at electrode Cz, superimposed for primed and unprimed target words in the pre-meal (**left panel**) and post-meal sessions (**right panel**) and for the Experimental and Control Group (**top vs**. **bottom panels**). Also shown are scalp topographies of the difference waves between ERPs to semantically unrelated and related targets words, displaying the distribution of the N400 in the time window 350 – 600 ms.

Figure 1 shows the ERPs at electrode Cz for primed and unprimed target words as a function of group and session and scalp topographies of the ERP difference between unprimed and primed target words. Relative to primed words, unprimed words elicited a long-lasting negativity starting shortly after 300 ms, reflecting the expected N400 effect. The ANOVA of the ERP amplitudes in the time window between 320–600 ms across all electrodes revealed sizeable priming effects in the N400 component, *F*(28,420) = 37.6, *p* < .001. This N400 effect increased from the pre-meal to the post-meal session, *F*(28,420) = 10.7, *p* < .001. Importantly, there was no modulation of the N400 by the meal situation nor any interactions, *F*s < 1.

Priming effects in performance were confined to error rates, which may relate to the relatively short prime-target interval. However, there was a standard N400 effect in ERP amplitudes, indicating normal semantic priming processes. None of these effects was modulated by factor group.

### Cognitive Control

#### Performance

Mean error rates ranged between 2.9 and 4.7% and were not affected by group or session. RTs for correct trials showed strong main effects of compatibility, *F*(1,15) = 146.4, *p* < .001, (*M*(comp. vs. incomp.) = 432 vs. 467 ms) and decreased from pre-meal to post-meal session (*M* = 462 vs. 437 ms), *F*(1,15) = 14.6, *p* < .01. Compatibility interacted significantly with session, *F*(1,15) = 11.9, *p* < .01, and with group, *F*(1,15) = 9.9, *p* < .01. Although there was no significant interaction between group, session, and compatibility, in line with our hypothesis the Simon effect in the EG seemed to be of comparable size in both sessions (Simon effect (incomp.-comp.): pre-meal = 43.8 ms, post-meal: 38.8 ms), whereas in the CG the effect was markedly smaller in the post-meal session (Simon effect: Pre-meal session = 38.8 ms, post-meal session = 24.6 ms). Therefore, we tested for compatibility by session interactions in each group which revealed a significant decline of the compatibility effect between pre- and post-meal session in the CG, *F*(1,15) = 21.7, *p* < .001, but not in the EG, *F*(1,15) = 2.2, *p* = .31.

The decline of the Simon effect in the CG across sessions indicates increased cognitive control in the post-meal session but might also be due to practice-induced overall faster RTs. In line with our hypothesis, however, for the EG the Simon effect was not modulated by session, indicating that the meal situation counteracted the reduction of interference that would normally come about with practice.

#### ERPs

**Figure 2:**
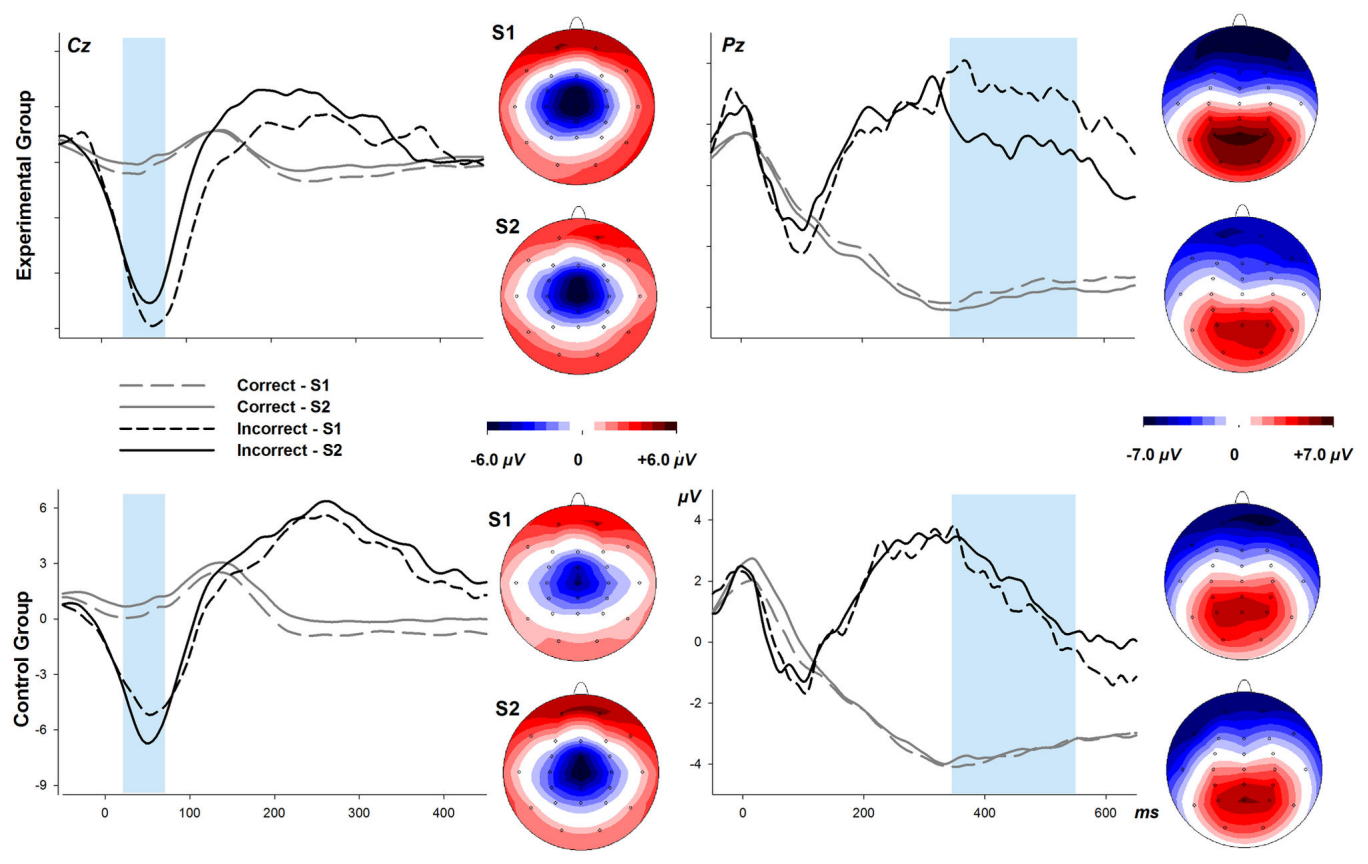
Response-synchronized ERPs from the Simon task. Left panel: ERPs at electrode Cz, superimposed for correct and incorrect (corr., incorr.) responses and pre-meal and post-meal sessions (S1, S2) and for the Experimental and Control group. Topographies of the Ne as the difference between incorrect and correct responses are depicted to the right of the waveforms. Right panel: Same as left panel but for electrode Pz (please note changes in voltage and time scales). Topographies of error positivities (350-550 ms) are shown to the right of the waveforms.

Of special interest were the ERPs elicited by errors in this task. One participant in the experimental group did not show any error responses in the Simon task and had to be dropped from this analysis. As shown in Figure 2 (left panels), following errors there was a fronto-centrally distributed negativity peaking around 50 ms – reflecting the Ne – which increased from the pre-meal to the post-meal session in the CG but declined in the EG. In line with this observation and the literature, the Ne amplitude was quantified as the average amplitude in the interval of 25 to 85 ms. The differential effect to be seen in the wave shapes was reflected in a trend for a four-way interaction of group, session, response correctness, and electrode, *F*(28,392) = 2.1, *p* = .088, when tested two-sided across all electrodes. The Ne in the present study was maximal at Cz; the amplitude difference between ERPs to errors and to correct trials tested at this electrode (where the Ne was largest) was highly significant, *F*(1,14) = 129.9, *p* < .001, and also showed a trend for an interaction between group, session, and correctness, *F*(1,14) = 3.8, *p* = .073. Post-hoc tests at the Cz electrode showed a trend towards an increase of the Ne in the CG, *F*(1,15) = 5.1, *p* < .08; but no change across sessions in the EG, *F*< 1.

Figure 2(right panels) shows that also the later part of the ERPs differed between correct and incorrect responses. The difference consisted in a parietal positivity starting around 350 ms, reflecting the error-related positivity (Pe). The amplitude of Pe diminished from pre-meal to post-meal session in the EG but not in the CG. When the Pe was quantified as mean amplitude between 350 and 550 ms and tested over all electrodes, the ANOVA revealed a significant 4-way interaction of group, session, response correctness, and electrode, *F*(28,392) = 2.4, *p* < .05. Post-hoc tests for each group showed that the reduction of the Pe was significant in the EG, *F*(28,392) = 3.6, *p* < .01, but there was no significant change in the CG (*F* = 1).

Together, the ERP analyses show that the Pe was reduced in the post-meal session as compared to the pre-meal session in the EG but not in the CG. Moreover, the CG showed a tentative increase of the Ne in the post-meal session whereas the EG did not – if anything, there was a numerical decline. Both findings are in line with our hypothesis that cognitive control is diminished by the restaurant meal. More specifically, our results indicate that this attenuation concerns aspects of response monitoring. In other words, after a good meal, errors loose some of their importance. This is not confined to higher order evaluative levels (Pe) but also seems to be present at a more automatic level like performance monitoring in errors (Ne).

### Emotion processing

#### Performance

Error rates varied between 8.0 and 9.6% and were unaffected by group or session. Reaction time for correct gender classifications were significantly affected by emotional expression, *F*(2,30) = 19.0, *p* < .001, with increased RTs for angry faces (*M* = 586 ms) as compared to happy and neutral faces (Ms = 569 and 578 ms, respectively).

#### ERPs

**Figure 3:**
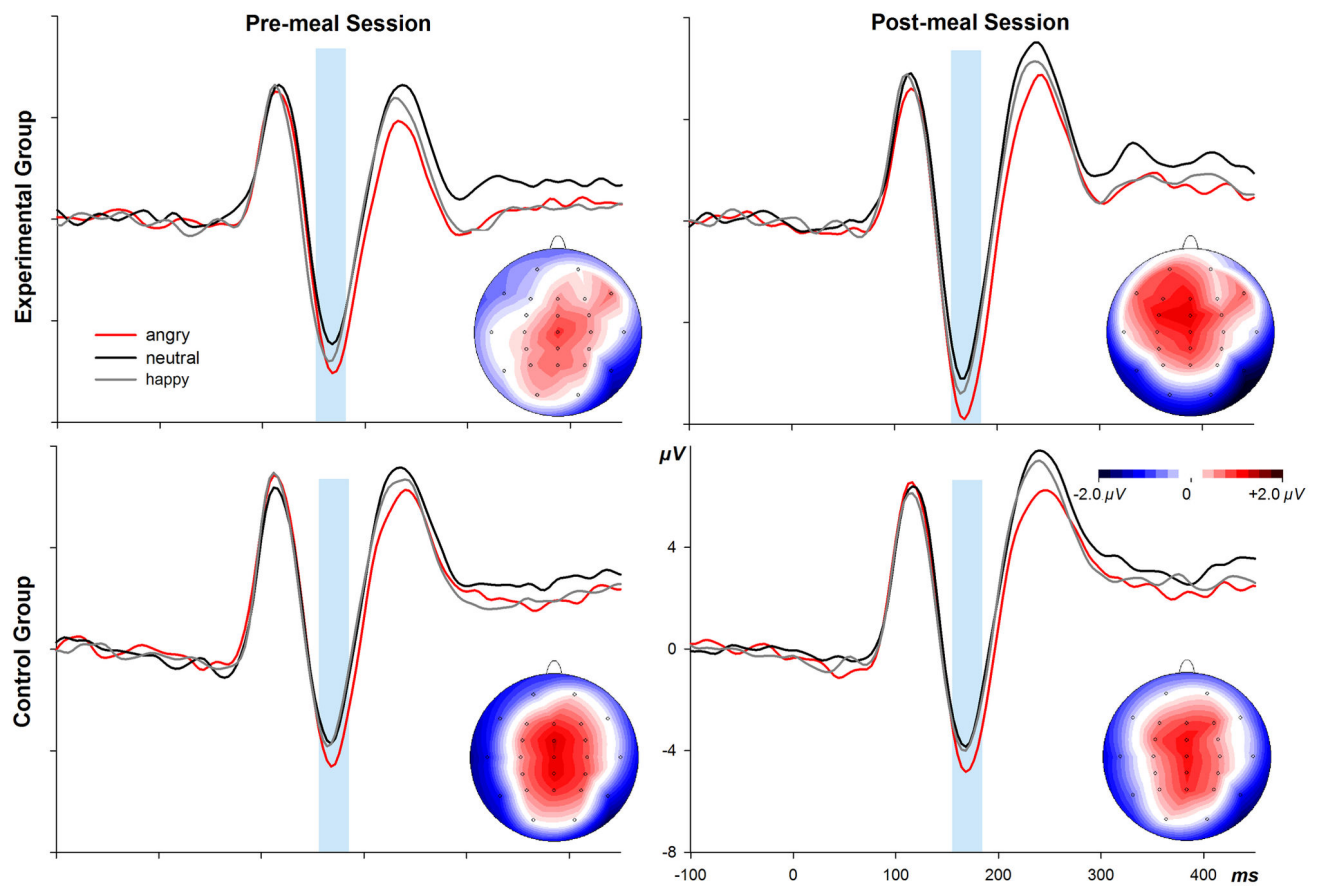
ERPs from the gender decision task at electrode P8, superimposed for the three facial expressions (**angry, neutral, happy**) in pre-meal and post-meal sessions and for the Experimental and Control group. Embedded heads depict scalp topographies of the difference waves between ERPs to angry and neutral facial expressions for the time range 160 to 180 ms.

Analysis of ERPs (Figure 3) proceeded in several steps. First, between 100 and 800 ms after the face presentation, mean amplitudes within 50-ms time windows were submitted to overall ANOVAs with factors group, session, emotion, and electrode (29 levels). This initial analysis was conducted because emotion effects can occur at rather different latencies and with different topographies [35, 32]. These analyses showed strong emotion x electrode interactions in all time segments, *F*s(56,840) > 3.5, ps ≤ .001. However, there were no interactions for emotion, group, session, and electrode as would be expected in terms of our research question. In line with current literature and because such approaches are less conservative than overall ANOVAs including all electrodes, we defined three regions of interest (ROI) including only those electrodes at which ERP effects of emotional expressions have shown their maximum amplitudes.

For the EPN, mean amplitudes in consecutive 50-ms steps between 100 and 400 ms on PO9 and PO10 electrodes (cf. [32, 35]) were analysed. Apart from replicating the strong emotion effects as obtained in the overall ANOVA, *F*s(2,30) > 6.6, *p*s < .01, there were no findings of interest. Next, we considered the LPC at Cz, CPz, and Pz electrodes (cf. [32]) between 400 and 650 ms in consecutive 50-ms steps. Again, there were no other effects apart from the strong emotion effects seen in the overall ANOVA, *F*s(2,30) > 4.7, *p*s < .05.

Finally, we targeted the N170 component at electrodes P7 and P8 (cf. [35]) at the peak latency of this component (mean amplitudes between 160 and 180 ms). ANOVA yielded a main effect of emotion, *F*(2,30) = 12.9, *p* < .01, with increasing amplitudes from neutral over happy to angry faces, *F*s(1,15) ≥ 8.6, *p*s < .05. Importantly, a 3-way interaction of factors group, session, and emotion, *F*(2,30) = 3.8, *p* < .05, was significant. Bonferroni-corrected post-hoc tests indicated a trend for an increase of negative emotion effects from pre-meal to post-meal session in the EG, *F*(1,15) = 5.4, *p* = .070, but not in the CG (*F* < 1). There were no effects for positive relative to neutral expressions.

Together, the restaurant meal seemed to enhance the response to negative emotional expressions at a rather early latency. Although early emotion effects at the latency of the N170 or even earlier are no exception, these findings are rather inconsistent and the boundary conditions for their emergence are poorly understood [35]. Nevertheless, it is very interesting that the meal manipulation enhances early analysis of negative facial expressions.

## General Discussion

In the present study, we compared the distal consequences of two different meal contexts on several cognitive and emotional processes. The experimental group (EG) dined in a restaurant in the company of a friend, could select their food, and had sufficient time to eat in leisure. In contrast, the control group (CG) consumed the same meals as their matched EG counterparts, but did so alone in a plain office environment without the opportunity to select their preferred meal and under time restrictions. Hence, we contrasted several context variables that had been shown to be important for food acceptability and emotions in previous research (see Introduction). In particular, we manipulated (at least) the following variables: social context (in the company of a person of preference vs. alone), availability of time (plenty vs. limited), service (being served vs. self-service), environment (spacious restaurant with music vs. a small, plain office with no music), control over food choice (choice from 20 dishes and 3 soft drinks vs. no choice), and a 15-minute walk after lunch vs. before lunch. The restaurant was chosen because of its modest pricing (affordable for students), while at the same time offering a tasty meal; that is, we compared two meal contexts that might be realistically implemented in daily routines. Because we contrasted two typical meal contexts, it is impossible to specify at this point, which of the variables above is/are crucial for the effects observed in our study. We want to argue, however, that although the relative contributions of our context variables to the observed effects are yet to be investigated, all of them are typically related – albeit in varying degrees – to meal situations. This holds true also for the walk, which commenced before or after the meal in the office and restaurant situations, respectively. Both the restaurant and the food were rated as good. Hence, our goal of providing a pleasant meal situation seems to have been accomplished. Future research should aim to isolate the contributions of the contextual factors to the effects observed in the present study and to be discussed next.

### Effects on Mood

Although the individual scales of the mood questionnaire did not show effects of the meal situation on mood, a post-hoc test of the combined RU and WM scales showed a significant change in the EG towards a calmer and less wakeful state after the meal, which was not present in the CG. Whereas these findings are admittedly post-hoc and not expected, they appear plausible, as they indicate that the restaurant meal situation had a relaxing effect as compared to the plain meal. Hence it seems that the restaurant meal indeed had an effect on the emotional state of the participants.

### Experimental effects

Importantly, the present study was not concerned with meal- or food-related effects but with the cognitive and affective consequences of meal context variations, the former being measured by means of several standard experimental tasks totally unrelated to the meal or food. We conducted three tasks in this regard, aiming at several cognitive and affective processes, which we assumed to be affected by meal-induced mood states.

#### Semantic Memory

Similar to previous findings [21], we had expected that the meal situation would diminish the N400 amplitude as measured using the semantic memory task, indicating facilitated semantic processes. In contrast to our expectations, we did not observe any modulation of the strong N400 elicited by our semantic relatedness manipulation. It is not clear why we did not find any effect on the N400; given that we did find effects in line with our predictions in the other tasks, we can rule out that our manipulation was ineffective. Maybe it is prudent to point out that previous reports on mood effects on N400 are scarce. Such effects, when found, have been observed under distinct conditions than those used in the current study. Federmeier et al. [21] obtained mood effects on N400 to unexpected words in sentences when they came from an unexpected semantic category – which during a neutral mood state elicited a very large N400 – but not when the unexpected words came from an expected category and elicited intermediate N400 amplitudes. In contrast, we presented semantically related and unrelated word pairs, which elicited a clear N400 of intermediate size. It is possible that semantic incongruence or unexpectedness must be relatively large to be amenable by mood. This would be in line with a recent study that found effect of discourse-induced emotion on syntactic but not on semantic processing at the sentence level [42].

#### Cognitive control

Cognitive control processes were studied in the Simon task. The Simon effect declined from the pre-meal to the post-meal session in the CG but not in the EG. This finding indicates that the restaurant situation counteracted the increasing efficiency of cognitive control with practice. ERP analysis could more precisely determine that response monitoring was diminished after the restaurant situation at least as concerns incorrect responses. This was observed both on a conscious level, as indicated by the error-related positivity, and on a preconscious level as indicated by the error-related negativity. These findings are in line with our assumption that positive mood as induced by the restaurant meal reduces cognitive control.

#### Emotion processing

The gender decision task about faces with different emotional expressions (Task 3) showed long-lasting and strong effects of facial emotional expressions as reflected by enhanced amplitudes of the N170, EPN, and LPC components. Interestingly, only at early stages of face processing (i.e., in the N170 component that is usually ascribed to the structural analysis of faces), were emotion effects enhanced by the meal manipulation if the expressions were threatening. In contrast, later emotion effects in the ERPs were unaffected by the meal manipulation. Along the lines of current thinking [43, 44], these findings can be interpreted as indicators that perceptual responsiveness to threatening stimuli at initial stages of face processing is facilitated by the state induced by the restaurant meal. This may seem surprising if one assumes that good mood after a nice meal might inoculate against aversive stimuli. However, if we consider the findings of diminished cognitive control from the Simon task, it may appear conceivable that the meal situation may lower one’s guards against negative stimuli.

### Conclusions and Perspectives

The findings of the present study, which was intended as an initial exploration into the effects of a typical daily meal situation, support our hypotheses in large part. Given the results of the combined analysis of the WM and RU mood scales, the restaurant meal – as compared to a plain office meal – had a relaxing effect at the conscious level, as reflected in subjective evaluations. The experimental effects in the post-meal sessions can be best summarized as a reduction of cognitive control on the performance level while the neurophysiological results indicate a slackening of cognitive control related to performance and error monitoring processes. Interestingly, the additional observation of increased ERP responses to threatening faces is consistent with a reduction of cognitive control. In a dual task study, Rellecke  [45] has shown that early effects of threat-related faces emerge when central cognitive resources are depleted by concurrent face-unrelated tasks. Such findings align with Pessoa’s [46] dual competition framework that assumes a challenge between emotional and cognitive control processing resources already at early perceptual stages. Accordingly, affectively significant events like threatening faces receive increased perceptual processing resources when cognitive control is reduced. Hence, we conclude that the restaurant meal – but not the office meal – appears to be relaxing and seems to reduce cognitive control processes for a while.

One may have expected more “positive” effects on the psychological processes of the participants who ate in the restaurant situation. However, the attenuation of cognitive control may be negative for certain purposes but not all. For example, reduced cognitive control is a disadvantage when close self-monitoring of performance and detailed attention to errors is required, such as in laboratory and factory work or numerical processing. In other situations, an attenuation of cognitive control may be advantageous, such as when social harmony or creativity is desired.

The present findings provide support for future studies into the effects of an overwhelmingly common situation. These studies might aim to (1) isolate the effective components of meal situations; (2) more broadly sample cognitive and affective meal effects; (3) specify interactions of the situational and food characteristics, and (4) investigate the neurocognitive mechanisms mediating the meal effects. Findings from these studies may be of great relevance for the understanding and the deliberate selection and - eventually – design of specific meal situations in restaurants and canteens in institutions as diverse as schools, universities, factories, hospitals, military, correctional institutions, or holiday resorts, depending on the overarching goal of these institutions. Hence, quite different meal situations may be optimal if the aim is cognitive control and exactness or if wellbeing and recreation is desired.

## References

[1] Flammang JA (2009) The taste for civilization. Food, politics, and civil society. Urbana: University of Illinois Press.

[2] Jones M (2007) Feast. Why humans share food. Oxford: OUP.

[3] Rozin P (2005) The meaning of food in our lives: A cross-cultural perspective on eating and well-being. J Nutr Educ Behav 37: 107-112. doi:10.1016/S1499-4046(06)60209-1. PubMed: 16246277. 10.1016/s1499-4046(06)60209-1 16246277

[4] Rozin P (1996) Towards a psychology of food and eating: From motivation to module to model to marker, morality, meaning, and metaphor. Curr Dir Psychol Sci 5: 18-24. doi:10.1111/1467-8721.ep10772690.

[5] Meiselman HL (2008) Experiencing products within a physical and social context. In Schifferstein HN J, Hekkert P (2008) Product Experience. Oxford: Elsevier pp. 559–580.

[6] Boutrolle I, Delarue J, Arranz D, Rogeaux M, Koster EP (2007) Central location test vs. home use test: Contrasting results depending on product type. Foods Qual Prefer 18: 490-499. doi:10.1016/j.foodqual.2006.06.003.

[7] Cardello AV, Bell R, Kramer FM (1996) Attitudes of consumers toward military and other institutional foods. Foods Qual Prefer 7: 7-20. doi:10.1016/0950-3293(95)00028-3.

[8] Edwards JSA, Meiselman HL, Edwards A, Lesher L (2003) The influence of eating location on the acceptability of identically prepared foods. Foods Qual Prefer 14: 647-652. doi:10.1016/S0950-3293(02)00189-1.

[9] Meiselman HL, deGraaf C, Lesher LL (2000) The effects of variety and monotony on food acceptance and intake at a midday meal. Physiol Behav 70: 119-125. doi:10.1016/S0031-9384(00)00268-7. PubMed: 10978486. 10978486 10.1016/s0031-9384(00)00268-7

[10] Sobal J, Nelson MK (2003) Commensal eating patterns: a community study. Appetite 41: 181-190. doi:10.1016/S0195-6663(03)00078-3. PubMed: 14550316. 14550316 10.1016/s0195-6663(03)00078-3

[11] King SC, Weber AJ, Meiselman HL (2004) The effect of meal situation, social interaction, physical environment and choice on food acceptability. Foods Qual Prefer 15: 645-653. doi:10.1016/j.foodqual.2004.04.010.

[12] Danziger S, Levav J, Avnaim-Pesso L (2011) Extraneous factors in judicial decisions. Proc Natl Acad Sci U S A 108: 6889-6892. doi:10.1073/pnas.1018033108. PubMed: 21482790. 21482790 10.1073/pnas.1018033108 PMC3084045

[13] Kniffin KM, Wansink B (2012) It’s not just lunch: Extra-pair commensality can trigger sexual jealousy. PLOS ONE 7: e40445. doi:10.1371/journal.pone.0040445. PubMed: 22792327. 22792327 10.1371/journal.pone.0040445 PMC3394702

[14] Ashby FG, Isen AM, Turken U (1999) A neuropsychological theory of positive affect and its influence on cognition. Psychol Rev 10: 529-550. doi:10.1037/0033-295X.106.3.529. 10.1037/0033-295x.106.3.529 10467897

[15] Dolan RJ (2002) Emotion, cognition, and behavior. Science 298: 1191-1194. doi:10.1126/science.1076358. PubMed: 12424363. 12424363 10.1126/science.1076358

[16] Mitchell RLC, Phillips LH (2007) The psychological, neurochemical and functional neuroanatomical mediators of the effects of positive and negative mood on executive functions. Neuropsychologia 45: 617-629. doi:10.1016/j.neuropsychologia.2006.06.030. PubMed: 16962146. 16962146 10.1016/j.neuropsychologia.2006.06.030

[17] Isen AM, Daubman KA, Nowicki GP (1987) Positive affect facilitates creative problem-solving. J Pers Soc Psychol 52: 1122-1131. doi:10.1037/0022-3514.52.6.1122. PubMed: 3598858. 3598858 10.1037//0022-3514.52.6.1122

[18] Baas M, De Dreu CKW, Nijstad BA (2008) A meta-analysis of 25 years of mood-creativity research: Hedonic tone, activation, or regulatory focus? Psychol Bull 134: 779-806. doi:10.1037/a0012815. PubMed: 18954157. 18954157 10.1037/a0012815

[19] Bless H, Fiedler K (1995) Affective states and the influence of activated general knowledge. Pers Soc Psychol Bull 21: 766-778. doi:10.1177/0146167295217010.

[20] Lyubomirsky S, King L, Diener E (2005) The benefits of frequent positive affect: Does happiness lead to success? Psychol Bull 131: 803-855. doi:10.1037/0033-2909.131.6.803. PubMed: 16351326. 16351326 10.1037/0033-2909.131.6.803

[21] Federmeier KD, Kirson DA, Moreno EM, Kutas M (2001) Effects of transient, mild mood states on semantic memory organization and use: an event-related potential investigation in humans. Neurosci Lett 305: 149-152. doi:10.1016/S0304-3940(01)01843-2. PubMed: 11403927. 11403927 10.1016/s0304-3940(01)01843-2

[22] Stürmer B, Nigbur R, Schacht A, Sommer W (2011) Reward and punishment effects on error processing and conflict control. Front Psychol 2: 335-. doi:10.3389/fpsyg.2011.00335. PubMed: 22110464. 22110464 10.3389/fpsyg.2011.00335 PMC3217218

[23] West R, Travers S (2008) Tracking the temporal dynamics of updating cognitive control: An examination of error processing. Cereb Cortex 18: 1112-1124. doi:10.1093/cercor/bhm142. PubMed: 17716989. 17716989 10.1093/cercor/bhm142

[24] Larson MJ, Kaufman DA, Kellison IL, Schmalfuss IM, Perlstein WM (2009) Double jeopardy! The additive consequences of negative affect on performance-monitoring decrements following traumatic brain injury. Neuropsychology 23: 433-444. doi:10.1037/a0015723. PubMed: 19586208. 19586208 10.1037/a0015723

[25] Kutas M, Federmeier KD (2011) Thirty years and counting: Finding meaning in the N400 component of the event-related brain potential (ERP). Annu Rev Psychol 62: 621-647. doi:10.1146/annurev.psych.093008.131123. PubMed: 20809790. 20809790 10.1146/annurev.psych.093008.131123 PMC4052444

[26] Falkenstein M, Hoormann J, Christ S, Hohnsbein J (2000) ERP components on reaction errors and their functional significance: a tutorial. Biol Psychol 51: 87-107. doi:10.1016/S0301-0511(99)00031-9. PubMed: 10686361. 10686361 10.1016/s0301-0511(99)00031-9

[27] van Steenbergen H, Band GPH, Hommel B (2009) Reward counteracts conflict adaptation. Psychol Sci 20: 1473-1477. doi:10.1111/j.1467-9280.2009.02470.x. PubMed: 19906127. 19906127 10.1111/j.1467-9280.2009.02470.x

[28] van Steenbergen H, Band GPH, Hommel B (2010) In the mood for adaptation: How affect regulates conflict-driven control. Psychol Sci 21: 1629-1634. doi:10.1177/0956797610385951. PubMed: 20943936. 20943936 10.1177/0956797610385951

[29] Leuthold H, Sommer W (1999) ERP correlates of error processing in spatial S-R compatibility tasks. Clin Neurophysiol 110: 342-357. doi:10.1016/S1388-2457(98)00058-3. PubMed: 10210624. 10210624 10.1016/s1388-2457(98)00058-3

[30] Falkenstein M, Hohnsbein J, Hoormann J, Blanke L (1991) Effects of crossmodal divided attention on late Erp components. 2. Error processing in choice reaction tasks. Electroen Clin Neuro 78: 447-455. doi:10.1016/0013-4694(91)90062-9. 10.1016/0013-4694(91)90062-9 1712280

[31] Junghöfer M, Sabatinelli D, Bradley MM, Schupp HT, Elbert TR et al. (2006) Fleeting images: rapid affect discrimination in the visual cortex. Neuroreport 17: 225-229. doi:10.1097/01.wnr.0000198437.59883.bb. PubMed: 16407776. 16407776 10.1097/01.wnr.0000198437.59883.bb

[32] Schacht A, Sommer W (2009) Emotions in word and face processing: Early and late cortical responses. Brain Cogn 69: 538-550. doi:10.1016/j.bandc.2008.11.005. PubMed: 19097677. 19097677 10.1016/j.bandc.2008.11.005

[33] Schupp HT, Flaisch T, Stockburger J, Junghöfer M (2006) Emotion and attention: event-related brain potential studies. Prog Brain Res 156: 31-51. doi:10.1016/S0079-6123(06)56002-9. PubMed: 17015073. 17015073 10.1016/S0079-6123(06)56002-9

[34] Recio G, Sommer W, Schacht A (2011) Electrophysiological correlates of perceiving and evaluating static and dynamic facial emotional expressions. Brain Res 1376: 66-75. doi:10.1016/j.brainres.2010.12.041. PubMed: 21172314. 21172314 10.1016/j.brainres.2010.12.041

[35] Rellecke J, Sommer W, Schacht A (2012a) Emotion effects on the N170: A question of reference? Brain Topogr 26: 62-71. doi:10.1007/s10548-012-0261-y. PubMed: 23053603. 23053603 10.1007/s10548-012-0261-y

[36] Oldfield RC (1971) The assessment and analysis of handedness: The Edinburgh inventory. Neuropsychologia 9: 97-113. doi:10.1016/0028-3932(71)90067-4. PubMed: 5146491. 5146491 10.1016/0028-3932(71)90067-4

[37] Hautzinger M, Keller F, Kühner C (2006) *Beck Depressions-Inventar* (BDI-II).. Revision Frankfurt/Main: Harcourt Test Services.

[38] Steyer R, Schwenkmezger P, Notz P, Eid M (1997) Der Mehrdimensionale Befindlichkeitsfragebogen (MDBF). Göttingen: Hogrefe.

[39] Dimigen O, Kliegl R, Sommer W (2012) Trans-saccadic parafoveal preview benefits in fluent reading: A study with fixation-related brain potentials. NeuroImage 62: 381–393. doi:10.1016/j.neuroimage.2012.04.006. PubMed: 22521255. 22521255 10.1016/j.neuroimage.2012.04.006

[40] Rellecke J, Palazova M, Sommer W, Schacht A (2011) On the automaticity of emotion processing in words and faces: Event-related brain potentials evidence from a superficial task. Brain Cogn 77: 23-32. doi:10.1016/j.bandc.2011.07.001. PubMed: 21794970. 21794970 10.1016/j.bandc.2011.07.001

[41] Rellecke J, Sommer W, Schacht A (2012b) Does processing of emotional facial expressions depend on intention? Time-resolved evidence from event-related brain potentials. Biol Psychol 90: 23-32. doi:10.1016/j.biopsycho.2012.02.002. 22361274 10.1016/j.biopsycho.2012.02.002

[42] Jiménez-Ortega L, Martín-Loeches M, Casado P, Sel A, Fondevila S et al. (2012) How the emotional content of discourse affects language comprehension. PLOS ONE 7: e33718. doi:10.1371/journal.pone.0033718. PubMed: 22479432. 22479432 10.1371/journal.pone.0033718 PMC3315581

[43] Ohman A, Mineka S (2001) Fears, phobias, and preparedness: Toward an evolved module of fear and fear learning. Psychol Rev 108: 483-522. doi:10.1037/0033-295X.108.3.483. PubMed: 11488376. 11488376 10.1037/0033-295x.108.3.483

[44] Vuilleumier P, Pourtois G (2007) Distributed and interactive brain mechanisms during emotion face perception: Evidence from functional neuroimaging. Neuropsychologia 45: 174-194. doi:10.1016/j.neuropsychologia.2006.06.003. PubMed: 16854439. 16854439 10.1016/j.neuropsychologia.2006.06.003

[45] Rellecke J (2012) Automaticity in emotional face processing [PhD dissertation]. Berlin: Department of Psychology , Humboldt-University . pp. 37 http://edoc.hu -berlin.de/dissertationen/rellecke-julian-2012-10-30/PDF/rellecke.pdf.

[46] Pessoa L (2009) How do emotion and motivation direct executive control? Trends Cogn Sci 13: 160–166. doi:10.1016/j.tics.2009.01.006. PubMed: 19285913. 19285913 10.1016/j.tics.2009.01.006 PMC2773442

